# Herpes virus entry mediator licenses *Listeria* infection induced immunopathology through control of type I interferon

**DOI:** 10.1038/srep12954

**Published:** 2015-08-06

**Authors:** Mengjie Lv, Weiwei Wu, Yuejiao Zhang, Mingzhao Zhu

**Affiliations:** 1 Key Laboratory of Infection and Immunity, Institute of Biophysics, Chinese Academy of Sciences, Beijing 100101, China; 2 University of Chinese Academy of Sciences, Beijing 100049, China

## Abstract

Apoptosis of the splenic lymphocytes is often induced during the acute phase of *Listeria* infection in mice. However, the underlying mechanism remains incompletely understood. Here, we found that herpes virus entry mediator (HVEM) plays an important role for *Listeria* infection induced lymphocyte apoptosis. Mechanistically, HVEM is not directly involved in listeriolysin O (LLO) induced lymphocyte apoptosis or interferon beta induced T cell activation per se. Interestingly, HVEM is partially required for *Listeria* induced interferon (IFN)-I production in the spleen, particularly in macrophages. Consequently, the bystander activation of lymphocytes is significantly lower in HVEM deficient mice than that in wild-type (WT) mice upon *Listeria* infection. Thus, our results have revealed a novel role of HVEM on the regulation of IFN-I and immunopathology during *Listeria* infection.

Infection induced immunopathological injury is a common health risk for humans. The interplay between microbes and the host has been an intriguing topic in the field. *L. monocytogens*, a Gram-positive facultative intracellular bacterium, causes severe diseases in immunocompromised hosts^1^. Studies in murine models demonstrated acute and dramatic immunopathological injury of the lymphoid compartment in the spleen upon *L. monocytogens* infection^2^. The pathological lesion could occur as early as 24 h after infection and peaks around 48 h. Depending on the infection dose, the lesion is primarily found at T cell area and may extend to the B cell follicles^3^,^4^,^5^. Thus, *L. monocytogens* infection in mice represents an accessible and useful model to study the molecular and cellular mechanisms regulating the infection induced immunopathology.

Earlier studies have found that listeriolysin O (LLO), a member of the cholesterol-dependent cytolysin family of pore-forming proteins, is a major virulence factor of *Listeria*. Mainly known for its role in phagosome escape, LLO released extracellularly at infective foci can induce apoptosis of surrounding lymphocytes^6^. In addition, activated lymphocytes seem more susceptible to LLO induced apoptosis than resting cells^5^.

Induction of type I interferon (IFN-I) is a notable host innate response upon *Listeria* infection after their engulfment by phagocytic cells^7^,^8^. Production of IFN-I activates lymphocytes and sensitizes them to LLO induced apoptosis in a bystander way^9^. In line with this, deficiency of IFN-I receptor on lymphocytes rescues them from apoptosis and results in lower bacterial burden in the host^10^,^11^. Thus, *Listeria* seems to exploit host IFN-I response for its productive infection. How the host regulates *Listeria* induced IFN-I production remains largely unclear.

Herpes virus entry mediator (HVEM), a member of TNF receptor superfamily, was first identified as a mediator for entry of herpes simplex viruses (HSVs)^12^. Later work revealed that HVEM can act as both a receptor [for LIGHT (homologous to lymphotoxin, exhibits inducible expression, and competes with the glycoprotein D for HVEM, a receptor expressed by lymphocytes)] and a ligand [for BTLA (B and T cell attenuator) and CD160], therefore delivers bidirectional signals in different types of cells^13^. Engagement of HVEM by LIGHT has been shown to transduce stimulatory pathways in T cells, NK cells and monocytes^14^,^15^,^16^,^17^. Ligation of HVEM to BTLA delivers inhibitory signals in T, B and dendritic cells (DCs)^18^,^19^,^20^. HVEM binding to CD160 may transduce either inhibitory or activating signals, depending on the scenarios^21^,^22^. Therefore, HVEM and its binding partners have been demonstrated diversified roles in regulating both innate and adaptive immune responses.

HVEM pathways have been found playing important roles in different types of cells during bacterial infection. HVEM was reported to enhance the bactericidal activity of human monocytes and neutrophils against *Listeria* and *S. aureus*^17^. In the mucosal epithelium, HVEM is required for signal transducer and activator of transcription 3 (STAT3) activation and host defense against pathological bacteria^23^. In the adaptive immunity, HVEM has been found to be important for the survival of the antigen activated CD8^+^ T cells during *Listeria* infection^24^. Interestingly, BTLA signaling was recently found promoting *Listeria* proliferation in CD8^+^ DCs, which also favors for long term T cell response^25^. While these studies have focused on the role of HVEM or BTLA for host defense, their impact on the host injury remains less clear. Understanding the mechanisms is necessary for better protection of the host when facing the infection.

In this study, we have investigated the role of HVEM on *Listeria* induced immunopathology. At acute phase of *Listeria* infection, obvious protection against splenic lesions was found in *Hvem*^-/-^ hosts. Mechanistically, production of IFN-I in the spleen was found at significantly lower level in *Hvem*^-/-^ mice than in WT mice. Thus, our study reveals a novel role of HVEM in IFN-I response during *Listeria* infection induced immunopathology. HVEM may act as a new host factor tipping the balance of host defense and host injury during infection.

## Results

### HVEM deficiency alleviates the splenic lesion during acute phase of *Listeria* infection

**Figure 1:**
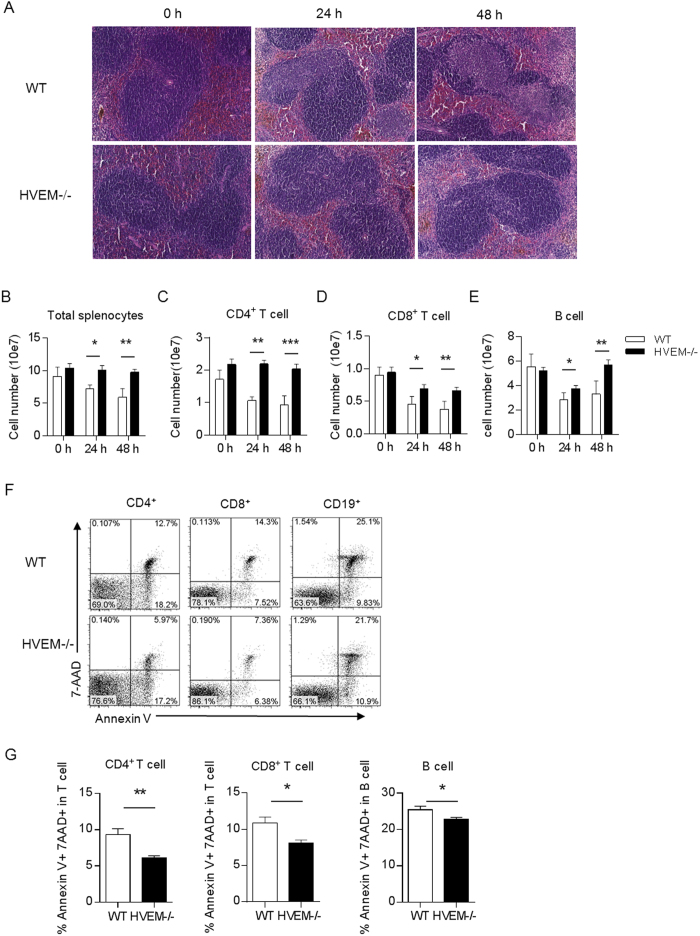
Alleviated spleen pathology in *Hvem*^-/-^ mice during *Listeria* infection. WT and *Hvem*^-/-^ mice were uninfected or infected i.p. with 1 × 10^7^ CFU of *Listeria*, 24 and 48 h after infection, spleens were examined by H&E (**A**), the total cellularity (**B**) and different subsets of lymphocytes (**C**–**E**) were counted. Original magnification is 20× for H&E staining. Error bar represents SEM. *P < 0.05; **P < 0.01; ***P < 0.001 (Student’s *t*-test). Data are representative from two experiments, n = 5 or 6 for each group in each experiment. (**F**,**G**) Mice were infected as described above, 24 h later, apoptosis of splenic lymphocytes was determined by Annexin V/7-AAD staining and FACS analysis. Data are pooled from two independent experiments, n = 5 or 6 for each group. Error bar represents SEM. *P < 0.05; **P < 0.01 (Student’s *t*-test).

To study the role of HVEM in *Listeria* induced splenic pathological change, WT and *Hvem*^-/-^ mice were infected i.p. with 10^7^ CFU of *L. Monocytogenes*. In WT mice, obvious splenic pathology was observed 24 h after infection and the lesion became severer at 48 h (Fig. 1A). In stark contrast, much less lesions were seen in the spleens from *Hvem*^-/-^ mice throughout the observation period (Fig. 1A). Consistent with the pathological change, the total cellularity of the spleen was dramatically decreased in WT mice upon infection, but largely maintained in *Hvem*^-/-^ mice (Fig. 1B). Flow cytometry analysis of different subsets of lymphocytes demonstrated significantly more loss of CD4^+^, CD8^+^ T cells and B cells in the spleens of WT mice compared to those in *Hvem*^-/-^ mice (Fig. 1C–E). To determine whether the lymphocytes of *Hvem*^-/-^ mice are protected from apoptosis, splenocytes were collected 24 h after infection and stained with apoptosis markers for flow cytometry analysis. Indeed, significantly lower rate of apoptosis of both CD4^+^ and CD8^+^ T cells and B cells were found in *Hvem*^-/-^ mice compared to those in WT mice (Fig. 1F,G). Thus, HVEM seems a detrimental host factor leading to lymphocyte apoptosis during acute *L. Monocytogenes* infection.

### HVEM is required for lymphocyte activation during *Listeria* infection

LLO can directly induce apoptosis of lymphocytes especially when lymphocytes are activated^5^. To determine whether HVEM expressed on lymphocytes intrinsically sensitizes them to LLO induced apoptosis, splenocytes from WT and *Hvem*^-/-^ mice were harvested and treated with live *L. Monocytogenes* with different ratios of bacteria to splenocytes. 24 h after *Listeria* treatment, significant apoptosis of T cells was found by flow cytometry analysis. However, no difference was found for apoptosis rate between WT and *Hvem*^-/-^ cells (Fig. S1). Therefore, it is unlikely that HVEM intrinsically regulates LLO induced lymphocyte apoptosis.

**Figure 2:**
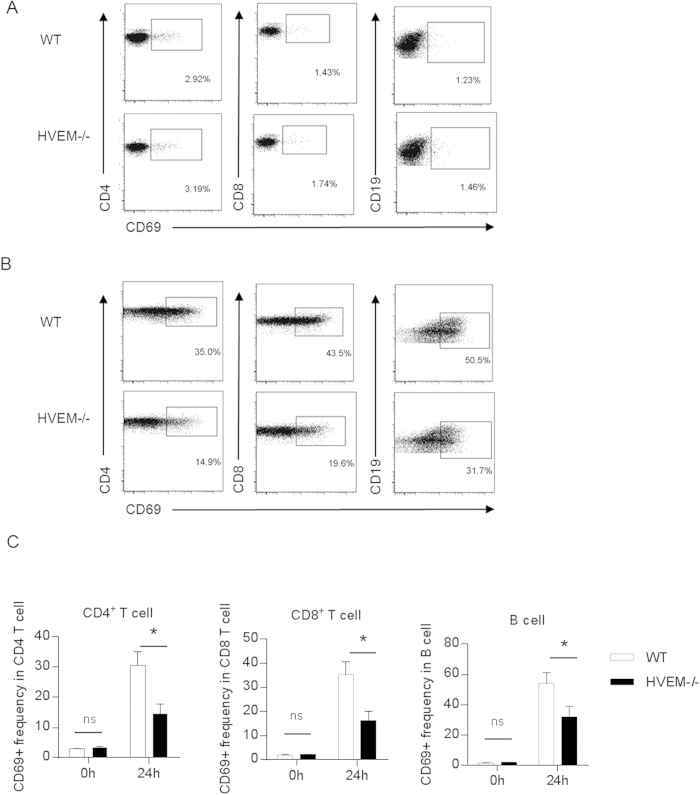
HVEM is required for lymphocyte activation during infection. WT and *Hvem*^-/-^ mice were infected as described above, 24 h later, splenic lymphocyte activation was determined by CD69 staining and FACS analysis with cells from uninfected mice as control. Representative FACS plots are shown in (**A** and **B**). Gate percentages indicate CD69^+^ cells. Statistical analysis is shown in (**C**). Data are pooled from two independent experiments, n = 7 or 8 for each group. Error bar represents SEM. *P < 0.05 (Student’s *t*-test).

Since activated lymphocytes are more susceptible to LLO induced apoptosis than resting cells and HVEM has been well documented as a costimulatory molecule for T cell activation, we asked whether HVEM may promote T cell bystander activation upon *Listeria* infection. To this end, WT and *Hvem*^-/-^ mice were infected with 10^7^ CFU of *L. Monocytogenes* as before. 24 h later, activation status of lymphocytes was assayed by staining the activation marker CD69 and flow cytometry analysis (Fig. 2A,B). While nearly 40% of T cells and 60% of B cells were positive for CD69 surface expression in WT mice 24 h after *Listeria* infection, dramatically fewer CD69^+^ lymphocytes were found in *Hvem*^-/-^ mice (Fig. 2C). The impaired lymphocyte activation found in *Hvem*^-/-^ mice is well consistent with the reduced apoptosis of lymphocytes.

### HVEM does not intrinsically regulate lymphocyte activation upon *Listeria* infection

**Figure 3:**
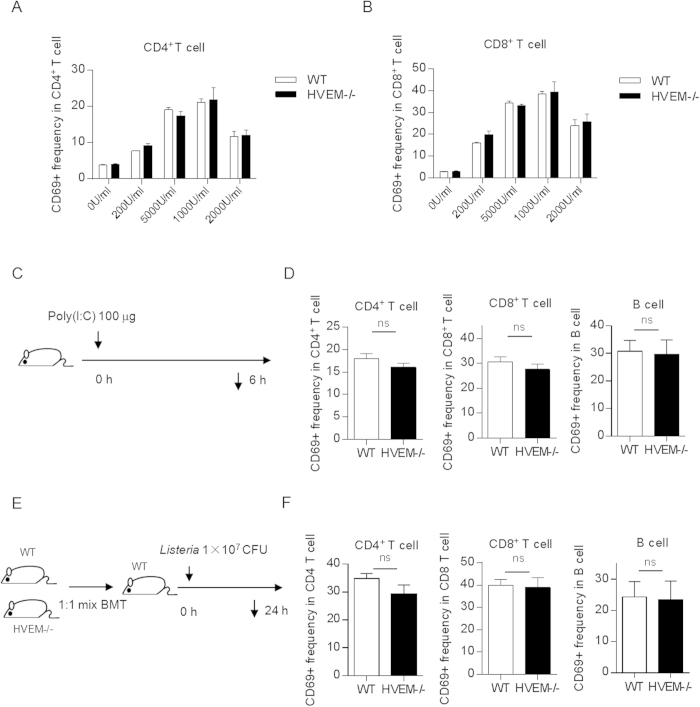
HVEM does not intrinsically regulate lymphocyte activation. (**A**,**B**) WT and *Hvem*^-/-^ splenocytes were treated with IFN-β of different concentrations overnight *in vitro*, the expression of CD69 on CD4^+^ (A) and CD8^+^ (**B**) T cells was measured by FACS. Error bar represents SEM. No significance was found between WT and *Hvem*^-/-^ groups (Student’s *t*-test). Data are representative from three experiments, n = 3 for each group in each experiment. (**C**) WT and *Hvem*^-/-^ mice were treated with 100 μg poly(I:C) i.v. as depicted. 6 h later, splenocytes were harvested, CD69 expression on different subsets of lymphocytes was measured by FACS (**D**). Data are pooled from two independent experiments, n = 5 or 6 for each group. Error bar represents SEM. ns, nonsignificant (Student’s *t*-test). (**E**) Established chimeric mice with mixed bone marrows (WT : *Hvem*^-/-^ = 1:1) were infected i.p. with 1 × 10^7^ CFU *Listeria* as depicted. 24 h later, CD69 expression on different subsets of splenic lymphocytes was measured by FACS (**F**). Data are representative of two independent experiments, n = 3 for each group in each experiment. Error bar represents SEM. ns, nonsignificant (Student’s *t*-test). The drawing of the mouse is made by M.Z.

IFN-I has been found critical for *Listeria* infection induced lymphocyte activation^26^. We asked whether HVEM is directly involved in IFN-I induced lymphocyte activation. However, neither *in vitro* stimulation by IFN-β nor *in vivo* stimulation by poly(I:C) revealed difference on activation between WT and *Hvem*^-/-^ lymphocytes (Fig. 3A–D). To further test whether HVEM intrinsically controls lymphocyte activation upon *Listeria* infection, bone marrow chimeric mice were generated with mixed bone marrow cells (with ratio of WT:HVEM = 1:1). 6–8 weeks after bone marrow transfer, chimeric mice were infected with *Listeria* and the lymphocyte activation was assayed as before (Fig. 3E). Comparable lymphocyte activation was found between WT and *Hvem*^-/-^ compartments (Fig. 3F). These data suggest that HVEM does not intrinsically regulate T cell activation during *Listeria* infection.

### HVEM is partially required for IFN-I production in splenic macrophages for lymphocyte activation and apoptosis

**Figure 4:**
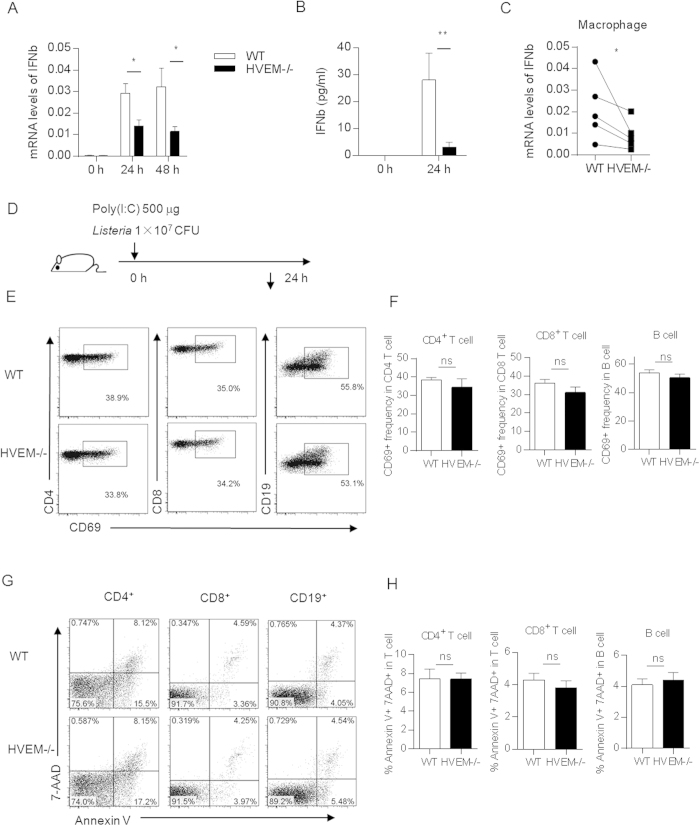
HVEM regulates IFN-I expression in splenic macrophages to enhance lymphocyte activation and apoptosis. (**A**,**B**) WT and *Hvem*^-/-^ mice were infected as described above, 24 and/or 48 h later, splenic expression of IFN-β was determined by qRT-PCR (A), IFN-β protein level in the sera was determined by ELISA (**B**). Samples from uninfected mice were also determined. Error bar represents SEM. *P < 0.05 (Student’s *t*-test). Data are pooled from three experiments for qRT-PCR assay, n = 6 to 8 for each group. For ELISA assay, data are representative from two independent experiments, n = 4 for each group in each experiment. (**C**) WT and *Hvem*^-/-^ mice were infected as above, 24 h later, splenocytes from mice of each group were pooled and the macrophages were sorted by flow cytometry as a sample. Expression of IFN-β was determined by qRT-PCR. Data are pooled from five independent experiments, n = 5 for each group. *P < 0.05 (paired Student’s *t*-test). (**D**) WT and *Hvem*^-/-^ mice were treated with 500 μg poly(I:C) i.v. and then infected with 1 × 10^7^ *Listeria* i.p. as depicted. (**E**,**F**) 24 h later, activation and apoptosis of splenic lymphocytes were determined as in Fig. 1. Representative FACS plots are shown in (**E** and **G**); statistical analysis is shown in (**F** and **H**). Data are representative of two independent experiments, n = 3 for each group in each experiment. Error bar represents SEM. ns, nonsignificant (Student’s *t*-test). The drawing of the mouse is made by M.Z.

The data described above indicate that HVEM may create an activating immune microenvironment for lymphocyte activation and/or apoptosis. IFN-I itself, as the most important activating factor for lymphocyte bystander activation during *Listeria* infection, was detected. IFN-I was rapidly induced upon *Listeria* infection in WT mice. Surprisingly, significantly lower IFN-I production was found in *Hvem*^*-/-*^ mice as early as 24 h post infection as determined by RT-PCR for the total spleen and ELISA for the sera (Fig. 4A,B). Furthermore, significantly lower expression level of IFN-I was also found in purified splenic macrophages but not in DCs (Fig. 4C and data not shown). The impaired production of IFN-I is unlikely due to reduced bacterial burden in the spleen of *Hvem*^-/-^ mice since comparable bacterial load was found between WT and *Hvem*^-/-^ mice (Fig. S2A). It is also unlikely due to difference of bacterial load at cellular level in the macrophages (Fig. S2B). Therefore, it seems that HVEM controls the IFN-I expression specifically in macrophages on a per cell basis. Given the complicated molecular interactions between HVEM and its binding partners and their broad expression profiles, the cellular and molecular basis for HVEM to regulate IFN-I expression remains an interesting question for future study.

To further test the causal relationship between the lower expression level of IFN-I and the alleviated lymphocyte activation and apoptosis in *Hvem*^-/-^ mice, mice were treated with poly(I:C) before *Listeria* infection (Fig. 4D). Poly(I:C) treatment induced comparable levels of IFN-I in both WT and *Hvem*^-/-^ mice (Fig. S3). Indeed, both lymphocyte activation (Fig. 4E,F) and apoptosis rate (Fig. 4G,H) were elevated in *Hvem*^-/-^ mice upon poly(I:C) treatment and reached to the level comparable to those in WT mice. Thus, HVEM deficiency does not confer lymphocytes inherent inability for activation upon IFN-I. IFN-I is downstream or independent of HVEM for the regulation of lymphocyte activation and apoptosis during *Listeria* infection.

### HVEM deficiency results in diminished inhibitory effect of *Listeria* clearance

**Figure 5:**
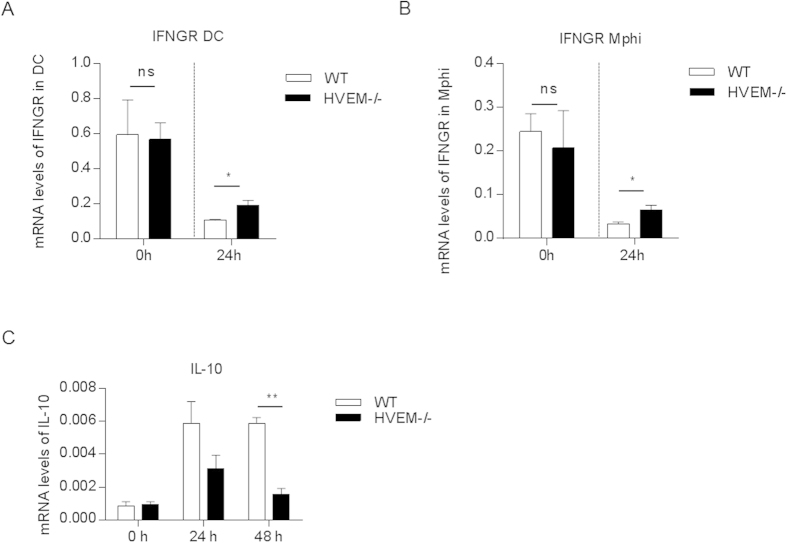
HVEM deficiency results in diminished inhibitory effect of *Listeria* clearance. (**A**,**B**) WT and *Hvem*^-/-^ mice were infected as above, 24 h later, splenocytes from mice of each group were pooled, DCs and macrophages were sorted by flow cytometry. Expression of IFNGR in DCs (**A**) and macrophages (**B**) were determined by qRT-PCR. Cells from uninfected mice were also determined. Data are pooled from three independent experiments, n = 3 or 4 for each group. *P < 0.05 (Student’s *t*-test). (**C**) WT and *Hvem*^-/-^ mice were infected as described above, 24 and 48 h later, mRNA level of IL-10 in spleen was determined by qRT-PCR. Data are representative of two experiments, n = 3 to 5 for each group in each experiment. Error bar represents SEM. **P < 0.01 (Student’s *t*-test).

IFN-I is a detrimental factor to the host during *Listeria* infection. IFN-I inhibits interferon gamma receptor (IFNGR) expression on innate cells thus limiting their activation induced by IFN-γ^27^. In addition, IFN-I sensitizes *Listeria* induced lymphocyte apoptosis, which upregulates anti-inflammatory cytokine IL-10 therefore dampening *Listeria* control^11^. We asked whether impaired IFN-I production in *Hvem*^-/-^ mice would result in rescued innate response, thus contribute to the *Listeria* resistance. We then examined IFNGR and IL-10 expression. Indeed, significantly higher level of IFNGR expression was found in *Hvem*^-/-^ mice on both DCs and macrophages compared to that in WT mice upon *Listeria* infection (Fig. 5A,B). In addition, IL-10 expression level was also significantly lower in the spleens of *Hvem*^-/-^ mice than that in WT mice upon *Listeria* infection (Fig. 5C). Together, these results provide new information how HVEM regulates innate immunity during *Listeria* infection.

## Discussion

IFN-I production is a common innate response during infections of numerous pathogens ranging from viruses, bacteria, parasites to fungi. Both protective and detrimental roles of IFN-I to the host have been reported^7^. Understanding how IFN-I production is regulated is an important topic in this field. Recent years have witnessed discoveries of increasing number of intracellular signaling molecules regulating IFN-I expression^28^. However, relatively little is known about its extracellular and intercellular regulation. Lymphotoxin beta receptor (LTβR), a member of TNF receptor superfamily has been reported to control IFN-I expression from fibroblasts during human or murine cytomegalovirus infection^29^,^30^. In addition, TNF has been also found to upregulate IFN-I from fibroblasts^31^. Whether other members of TNF receptor superfamily control IFN-I expression is unclear. In addition, whether IFN-I production from the immune cells could be regulated by members of TNF receptor superfamily remains elusive. Here, we have reported that HVEM is partially required for IFN-I expression in splenic macrophages upon *Listeria* infection.

Impaired expression of IFN-I in *Hvem*^-/-^ mice is likely a major contributing factor resulting in the lower level of lymphocyte activation and alleviated lymphocyte apoptosis. In line with this, increasing IFN-I production by poly(I:C) treatment enhanced lymphocyte activation and apoptosis rate in *Hvem*^-/-^ mice to levels comparable to those in WT mice. Furthermore, impaired IFN-I expression in *Hvem*^-/-^ mice leads to diminished IL-10 expression and higher IFNGR expression, which provide further information how HVEM deficiency confers the host resistant to *Listeria* infection.

Several types of innate cells including macrophages and TNF-iNOS producing dendritic cells (Tip-DCs) have been reported for IFN-I production upon *Listeria* infection^32^,^33^,^34^. In our study, IFN-β RNA expression was found in both macrophages (defined as CD11b^+^ F4/80^+^) and Tip-DCs (defined as ly6C^hi^CD11b^+^ CD11c^+^ MHC-II^+^), with about 10 folds higher level in the latter cell population. However, given the larger amount of macrophages than Tip-DCs (40 folds more at 24 h after infection), macrophages may be still the major contributor of IFN-β. In our study, we interestingly found that it is the macrophage but not TipDCs that showed impaired IFN-I production in the HVEM deficiency. The mechanism for the cell type selective regulation by HVEM remains to be determined.

The underlying cellular and molecular mechanisms for HVEM to control IFN-I expression during *Listeria* infection remain elusive. *Hvem*^-/-^ bone marrow derived macrophages seem to respond normally to *Listeria* stimulation compared with WT cells (data not shown). This suggests that HVEM does not intrinsically alter the capability of macrophage for IFN-I production induced by *Listeria*. More interestingly, our preliminary data indicate that HVEM may not function as a receptor for IFN-I regulation, since an agonistic anti-HVEM (14C1.1) does not promote *Listeria* induced IFN-I expression in macrophages (data not shown), although other stimuli or primary macrophages should be tested. Considering the complicated cellular interactions mediated by HVEM and its interacting partners, it would be intriguing to dissect which molecule (BTLA or CD160) interacts with HVEM, which cell interacts with macrophages and how the interaction works. Systemic comparison of the gene expression profile in macrophages from WT and *Hvem*^-/-^ mice may provide cues for the underlying intracellular mechanisms. Conditional knockout of HVEM, BTLA, or CD160 would be valuable tools to address the cellular interactions *in vivo*.

In conclusion, our study has demonstrated a novel important role of HVEM in the regulation of *Listeria* induced IFN-I production and immunopathology. This may open a new avenue to understand and control the IFN-I response not only for *Listeria* infection, but also for other pathogens.

## Methods

### Mice

*Hvem*^-/-^ mice were described previously^35^. WT C57BL/6 mice were purchased from Vital River laboratory Animal Technology Co. Beijing, China. All mice were housed under specific pathogen-free conditions in the animal care facilities at the Institute of Biophysics, Chinese Academy of Sciences. All animal experiments were performed in accordance with the guidelines of the Institute of Biophysics, Chinese Academy of Sciences, using protocols approved by the Institutional Laboratory Animal Care and Use Committee.

### *Listeria* infection, treatment and determination of CFU

The recombinant *Listeria* strain *Listeria*-OVA was described previously^36^ and provided by Dr. Honglin Xu (National Vaccine and Serum Institute, Beijing, China). *Listeria* was grown in brain-heart infusion broth with 5 μg/ml erythromycin. For determination of bacterial load in tissues, WT and *Hvem*^-/-^ mice were infected with 1 × 10^7^ *Listeria* by i.p. injection. At indicated time points after infection, organs were homogenized and lysed in sterile water with 0.5% Triton-100. Serial dilutions were plated on brain-heart infusion agar plates. Colonies were counted after incubation at 37 °C for 2 days. For poly(I:C) treatment, mice were injected i.v. with 100 or 500 μg poly(I:C) (Sigma) dissolved in sterile saline.

### Bone marrow transfer

Bone marrow chimeras were generated with 5 × 10^6^ bone marrow cells from donor mice transplanted i.v. into lethally irradiated congenic C57BL/6 host mice. To generate mixed bone marrow chimaeras, 2.5 × 10^6^ bone marrow cells were obtained from WT and *Hvem*^-/-^ mice, and mixed at a ratio of 1:1. Chimeras were given prophylactic water containing antibiotics and were analyzed 6–8 weeks after transplantation.

### Histology

Spleens were fixed in 4% paraformaldehyde and embedded in paraffin. Sections were stained with hemotoxylin eosin (H&E). Slides were scanned on Leica SCN400 F slide scanner.

### Flow Cytometry

The single cell suspension of spleen was prepared. The following antibodies were used for immunofluorescence staining: 7-AAD (BD Biosciences), F4/80(BM8), CD4(RM4-5), CD8(53-6.7), CD19(6D5), CD69(H1.2F3) all from eBioscience; Annexin V, CD11b(M1/70), CD11c(N418), Ly6C(HK1.4), MHC II(M5/114.15.2) all from Biolegend. Samples were acquired on BD LSRFortessa instrument and analyzed with FlowJo software. CD11b^+^ F4/80^+^ macrophages and Ly6C^+^ CD11b^+^ CD11c^+^ MHC II^+^ Tip-DCs were sorted on BD FACSAria III.

### ELISA

Mice were infected i.p. with *Listeria* or injected i.v. with 100 μg poly(I:C). Sera were collected 24 h after infection or 6 h after poly(I:C) treatment. The levels of IFN-β were determined using Mouse IFN-β ELISA kit with pre-coated plates (Biolegend). Plates were read at 450 nm using a SpectraMax Plus (Molecular Devices, Sunnyvale, CA, USA).

### Quantitative PCR

Total RNA from spleens was isolated with Trizol (Invitrogen). RNA from sorted cells was extracted by RNeasy Plus Mini Kit (Qiagen). RNA was digested with DNaseI and reverse transcribed into cDNA for real-time PCR. The levels of gene expression were normalized to β-actin. The following primers were used: β-actin, forward primer: 5′-ACACCCGCCACCAGTTCGC, reverse primer: 5′- ATGGGGTACTTCAGGGTCAGGGTCAGGATA; IFN-β, forward primer: 5′-CCATCCAAGAGATGCTCCAG, reverse primer: 5′-GTGGAGAGCAGTTGAGGACA; IFNGR, forward primer: 5′-CCTGTCGTATGCTGGGAATA, reverse primer: 5′-AATGTTGGTGCAGGAATCAG; IL-10, forward primer: 5′-CGCTGTCATCGATTTCTCC, reverse primer: 5′-ACACCTTGGTCTTGGAGCTT.

### Statistical Analysis

All data were analyzed using unpaired two-tail Student’s *t*-test, except the mRNA levels of sorted cells was analyzed by paired two-tail Student’s *t*-test. Analyses were performed using GraphPad Prism software (GraphPad Software Inc., San Diego, CA, USA). A value of P < 0.05 was considered statistically significant (*P < 0.05; **P < 0.01; and ***P < 0.001).

## Additional Information

**How to cite this article**: Lv, M. *et al.* Herpes virus entry mediator licenses *Listeria* infection induced immunopathology through control of type I interferon. *Sci. Rep.* **5**, 12954; doi: 10.1038/srep12954 (2015).

## Supplementary Material

Supplementary Information
