# Supplementary material for: Herpes virus entry mediator licenses Listeria infection induced immunopathology through control of type I interferon
Source: Sci Rep. 2015 Aug 6;5:12954. doi: 10.1038/srep12954 (PMC4526852; doi:10.1038/srep12954)
Supplement: Supplementary Information [file srep12954-s1.doc]

**Herpes virus entry mediator licenses *Listeria* infection induced immunopathology through control of type I interferon**

Mengjie Lv^1,2^, Weiwei Wu^1,2^, Yuejiao Zhang^1^ & Mingzhao Zhu^1*^

^1^ Key Laboratory of Infection and Immunity, Institute of Biophysics, Chinese Academy of Sciences, Beijing 100101, China.

^2^ University of Chinese Academy of Sciences, Beijing 100049, China.

Keywords: HVEM, Type I interferon, *Listieria*, Immunopathology

Correspondence: Dr. Mingzhao Zhu, Key Laboratory of Infection and Immunity, Institute of Biophysics, Chinese Academy of Sciences, #15 Datun Road, Chaoyang District, Beijing 100101, China. Fax: 86-10-64884618; Email: [zhumz@ibp.ac.cn](mailto:zhumz@ibp.ac.cn)

**Figure S1.**

**Figure S1. HVEM does not directly regulate *Listeria* induced lymphocytes apoptosis.**

WT and *Hvem*^‑/-^ splenocytes were treated by live *Listeria* with different ratios of bacteria:cell overnight *in vitro*. Cell apoptosis was measured by Annexin V/7-AAD staining and FACS analysis. The percentages of Annexin V/7-AAD double positive cells are shown. Data are pooled from four independent experiments, n=4 for each group. Error bar represents SEM. ns, nonsignificant (Student’s *t*-test).

**Figure S2.**

A

B

**Figure S2. The bacterial load in the spleen at early stage of *Listeria* infection.**

(A) WT and *Hvem*^‑/-^ mice were infected i.p. with 1×10^7^ CFU *Listeria*, at different time points (6, 24 and 48 h) after infection, splenic bacterial load was determined by colony assay. Error bar represents SEM, n=4 for each group. ns, nonsignificant (Student’s *t*-test). (B) WT and *Hvem*^‑/-^ mice were infected as above, 24 h later, splenocytes from mice of each group were pooled and the macrophages were sorted by flow cytometry as a sample. Expression of hly (encoding listeriolysin O) was determined by qRT-PCR. Data are pooled from five independent experiments, n=5 for each group. ns, nonsignificant (Student’s *t*-test).

**Figure S3.**

**Figure S3. Poly(I:C) treatment induces comparable levels of IFN-β production in WT and HVEM deficient mice.**

WT and *Hvem*^‑/-^ mice were treated i.p. with 100 μg poly(I:C). 24 h after treatment, mouse sera were collected, IFN-β protein was measured by ELISA. Data are pooled from two independent experiments. Error bar represents SEM, n=8 for each group. ns, nonsignificant (Student’s *t*-test).
